# Editorial: Phantom pain: mechanisms and updates in management

**DOI:** 10.3389/fpain.2025.1567956

**Published:** 2025-02-24

**Authors:** Weibin Shi, Balakrishnan Prabhakaran, Thiru Annaswamy

**Affiliations:** ^1^ Department of Physical Medicine and Rehabilitation, Penn State Health Milton S. Hershey Medical Center, Penn State College of Medicine, Hershey, PA, United States; ^2^ AI Plus Institute, University at Albany, Albany, NY, United States

**Keywords:** phantom pain management, phantom limb pain, neuropathic pain, peripheral mechanisms, modulation, mixed reality (MR), cognitive multisensory rehabilitation, mirror therapy (MT)

**Editorial on the Research Topic**
Phantom pain: mechanisms and updates in management

Phantom limb pain (PLP) is one of the most enigmatic and distressing conditions following limb loss, and it continues to present a significant challenge in both diagnosis and treatment. Despite considerable advancements in pain management, the precise mechanisms behind PLP remain elusive, which complicates the development of effective therapeutic strategies. While emerging technologies (e.g., mixed reality system) have overall shown promise in pain management, their application to PLP remains in the early stages of exploration (1).

In this special issue, *Phantom Pain: Mechanisms and Updates in Management*, the authors examine complex pathophysiologies, including phenomena such as phantom limb telescoping, risk factors associated with PLP development, and innovative treatment approaches like Cognitive Multisensory Rehabilitation (CMR). We trust that this issue will deepen the current understanding of PLP and shed light on the intricate experiences of individuals with limb loss and their management.

The case report by Aternali et al. presents a detailed case study that delves into the interplay between phantom limb telescoping and PLP which has not been well studied and illuminates several nuances that have not been previously elaborated. The investigation of telescoping—a phenomenon where the perceived size of the phantom limb diminishes during an episode—offers novel insights that could enhance our understanding of pain mechanisms post-amputation. While telescoping is usually considered to be phenomenon related to increased levels of phantom pain (2), this report implies that telescoping could be an adaptive form of plasticity that is accompanied by a reduction in PLP severity. By employing a longitudinal perspective and validated assessment tools, this study highlights the importance of psychosocial factors, such as resilience and mental health, in managing PLP. While the findings are rich in detail, the limitations of a single case study underscore the need for broader research to generalize these insights.

The potential of CMR as a treatment for PLP is compellingly illustrated in a case study by Zernitz et al. demonstrating its effectiveness, especially for patients resistant to conventional therapies. CMR is a physical therapy treatment that helps improve body awareness, movement, and sensation. CMR was used initially to treat patients with spinal cord injuries and other conditions that impact movement and sensation (3) Recently, CMR has been applied to chronic pain conditions, such as complex regional pain syndrome (4) By focusing on restoring mental body representations through multisensory exercises, CMR addresses the cortical remapping underlying PLP. This long-term follow-up reveals not only sustained pain relief but also an empowering self-management strategy for the patient. The single-case design limits broader applicability, and further investigation into the mechanisms of action and patient variability is essential.

The mini-review by Ishigami and Boctor provides a comprehensive overview of the risk factors contributing to PLP, categorizing them into pre-operative, intra-operative, and post-operative stages. This structured approach not only highlights the high prevalence of amputations and the projected increase in rates but also emphasizes the role of advanced surgical techniques, such as targetted muscle reinnervation (TMR). This mini-review also acknowledges that emotional and socio-economic factors play a critical role in PLP development, which has been addressed in more detail in this special issue in Wu et al.’s extensive review. The variability in reported PLP prevalence rates calls for further exploration to identify underlying factors that contribute to these discrepancies.

Wu et al.’s comprehensive review synthesizes relevant pathophysiologies, emphasizing the complexity of PLP through the lens of the central and peripheral nervous systems, psychosocial factors, and genetic influences. It successfully contextualizes the evolution of pain theories, narrating current understandings within a historical framework. By assimilating major human and animal studies, the authors deepen our understanding of this complex condition at a profound molecular level. The review advocates for a biopsychosocial model of care, highlighting the need for further research to clarify the mechanisms of PLP and improve clinical strategies. This inquisitive approach opens more avenues for future investigations and potential novel therapeutic approaches. We concur with the authors that PLP is likely attributable to a complex pathophysiology, necessitating interdisciplinary management.

The collective insights from these articles underscore the necessity of a holistic approach to manage PLP. They highlight the interplay between physical, psychological, and social factors, suggesting that effective pain management requires addressing all these dimensions. Moreover, every individual experiencing PLP with unique presentation likely has different underlying pathophysiologies as listed in Wu et al.’s review. As we look to the future, the call for larger, controlled studies is paramount to validate these findings and establish best practices which are based on reliable and sound evidence. By integrating individualized innovative treatments, understanding risk factors, and fostering interdisciplinary collaboration, we can better support individuals navigating the complex landscape of phantom limb pain, ultimately enhancing their quality of life.
